# Modification of Mechanical Properties, Polymerization Temperature, and Handling Time of Polymethylmethacrylate Cement for Enhancing Applicability in Vertebroplasty

**DOI:** 10.1155/2016/7901562

**Published:** 2016-10-12

**Authors:** Ching-Lung Tai, Po-Liang Lai, Wei-De Lin, Tsung-Tin Tsai, Yen-Chen Lee, Mu-Yi Liu, Lih-Huei Chen

**Affiliations:** ^1^Department of Mechanical Engineering, Graduate Institute of Medical Mechatronics, Chang Gung University, Kweishan, Taoyuan, Taiwan; ^2^Department of Orthopedic Surgery, Bone and Joint Research Center, Chang Gung Memorial Hospital, Chang Gung University College of Medicine, Kweishan, Taoyuan, Taiwan

## Abstract

Polymethylmethacrylate (PMMA) bone cement is a popular bone void filler for vertebroplasty. However, the use of PMMA has some drawbacks, including the material's excessive stiffness, exothermic polymerization, and short handling time. This study aimed to create an ideal modified bone cement to solve the above-mentioned problems. Modified bone cements were prepared by combining PMMA with three different volume fractions of castor oil (5%, 10%, and 15%). The peak polymerization temperatures, times to achieve the peak polymerization temperature, porosities, densities, modulus and maximum compression strengths of standard (without castor oil), and modified cements were investigated following storage at ambient temperature (22°C) or under precooling conditions (3°C). Six specimens were tested in each group of the aforementioned parameters. Increasing castor oil content and precooling treatment effectively decreased the peak polymerization temperatures and increased the duration to achieve the peak polymerization temperature (*P* < 0.05). Furthermore, the mechanical properties of the material, including density, modulus, and maximum compression strength, decreased with increasing castor oil content. However, preparation temperature (room temperature versus precooling) had no significant effect (*P* > 0.05) on these mechanical properties. In conclusion, the addition of castor oil to PMMA followed by precooling created an ideal modified bone cement with a low modulus, low polymerization temperature, and long handling time, enhancing its applicability and safety for vertebroplasty.

## 1. Introduction

Osteoporosis is common in aging populations. In the US, the prevalence of osteoporosis is 10.3% in adults 50 years and older; women in the same age group have a higher prevalence at 15.4% [1]. The rate of compression fractures is 20% in people 70 years and older and 16% in postmenopausal women [2]. A study by Johnell and Kanis also found that osteoporosis causes more than 8.9 million fractures each year and that osteoporotic fractures occur every 3 seconds [3]. Thus, it is very important to determine how to treat and prevent osteoporotic vertebral compression fractures. In general, vertebroplasty is suggested to treat vertebral compression fractures to increase the rigidity, supporting force and recovery height of the collapsed spinal vertebrae.

Vertebroplasty is a well-established, common treatment for acute osteoporotic vertebral compression fractures. Vertebroplasty can reduce pain and allows for rapid rehabilitation [4–6]; however, secondary vertebral compression fractures after vertebroplasty with polymethylmethacrylate (PMMA) bone cement augmentation often occur [7], at rates ranging from 12% to 52% [8, 9]. The inherent characteristics of PMMA, such as its excessive stiffness, exothermic polymerization, and short handling time, are considered the main factors leading to surgery failure, particularly for patients with osteoporosis [9–12].

PMMA bone cement is widely used in vertebroplasty because of its low cost and high stability. However, PMMA has various disadvantages. First, PMMA may cause thermal injury [13, 14], as PMMA polymerizes via an exothermic reaction that can cause necrosis in tissues close to the treatment location. Second, PMMA has high Young's modulus (*E*) and high compressive strength (*σ*_*c*_). The Young modulus of PMMA ranges from 2,000 to 3,000 MPa, which is much higher than the Young modulus of spongy bone, which ranges from 50 to 800 MPa [15, 16]. This large difference in material properties increases the risk of secondary fracture [17, 18]. Finally, the handling time of PMMA is short and may be not sufficiently long for clinical use. This is a dangerous factor for patients. In recent years, calcium phosphate cement (CPC) has been designed to resolve the above-listed limitations of PMMA [19]. CPC has low *E*, low *σ*_*c*_, low reacting temperature, and established biological activities [20, 21]; however, the lower initial *E* and the absorption of CPC can lead to other issues. For instance, the initial mechanical strength of CPC may be not sufficient for some osteoporosis cases [22]. The absorption of CPC may lead to collapse of the augmented vertebrae. CPC is also less economical than PMMA and suffers from a lack of clinical studies; thus, methods of improving PMMA for use in hospitals and as a biomaterial are needed.

Various methods of improving PMMA have been reported. It has been demonstrated that the addition of castor oil to PMMA can change its mechanical properties by lowering its Young's modulus, compressive strength, and reacting temperature [23, 24]. In our recent study [25], precooling raw PMMA material effectively slowed its polymerization reaction and thus lengthened its handling time in vertebroplasty [25]. Precooling and the addition of castor oil are simple and inexpensive methods of enhancing the applicability of PMMA for clinical settings. However, how PMMA changes following such treatments is currently unknown. Therefore, in the current study, two groups of PMMA samples were investigated: one stored at room temperature and the other stored under precooling conditions. Each group was composed of four different PMMA sample types, created by mixing PMMA with different volumes of castor oil.

## 2. Materials and Methods

### 2.1. Sample Preparation

In this study, the commercially available acrylic bone cement Simplex® P (Stryker, Kalamazoo, MI, USA) and castor oil (Hubei Ketian Pharmaceutical Co., Taiwan) were employed. The package of bone cement is composed of 40 g PMMA polymer powder and 20 cc liquid monomer. PMMA samples were divided into two major groups: a normal temperature group (NTG) and a precooling group (PCG). In the NTG, PMMA polymer powder and liquid monomer were maintained at 22°C for 24 hours; in the PCG, they were maintained at 3°C for 24 hours through the use of thermostatic controlling equipment. The two major groups were then divided into four subgroups: one control group and three experimental groups. In the control group, the PMMA polymer powder and liquid monomer were mixed for 1 minute, and no castor oil was added. This group was designated “M0.” For the experimental groups, liquid-phase PMMA samples were mixed with castor oil at 5%, 10%, and 15% (wt%) volumes and denoted as “M5,” “M10,” and “M15,” respectively. A maximum content of castor oil of 15 wt% was chosen because, in our pilot study, we found it difficult to achieve a uniform mixing of castor oil when the content of castor oil was up to 20 wt%. This leads to a severely uneven distribution of porosities and also difficulty for injection of the mixture. Unevenly distributed porosity may provide imbalanced support to a vertebral body after vertebroplasty, which may increase the risk of refracture at the weaker side. In the experimental groups, the PMMA polymer powder and liquid monomer were roughly mixed prior to the addition of castor oil. Then, the mixture was blended with castor oil for 1 minute, and the viscosity was measured for 10 seconds after waiting for 1 minute [26]. All of the samples were then divided into eight groups: NTG-M0, NTG-M5, NTG-M10, NTG-M15, PCG-M0, PCG-M5, PCG-M10, and PCG-M15. Blending cement was added to a forming syringe, and the samples were allowed to solidify for 48 hours [27]. Following this, the solidified samples were cut into cylinders with a diameter of 13 mm and a height of 26 mm (by ASTM D695 standard), after which they were subjected to three tests. The size of each sample was verified using Vernier calipers (Mitutoyo, 200 mm/0.02 mm), and a precision balance (HXB 300 g/0.01 g) was used to measure the weight of each sample. Then, the density of each sample was calculated.

### 2.2. Porosity Observation

**Figure 1:**
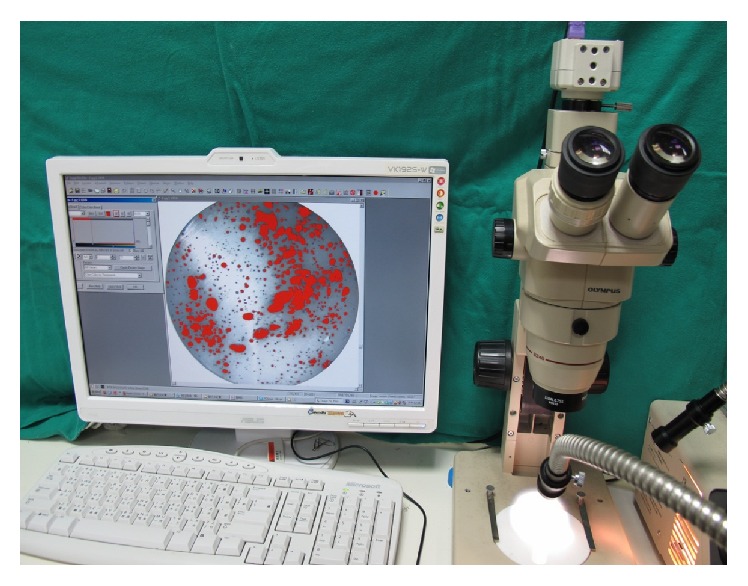
Photograph showing the porosity observation. Cavities on the sample surface were observed using an optical microscope. Following an image of the sample was captured, the image was analyzed using Image-Pro Plus 7.0 software.

Six samples in each subgroup were used to assess porosity. Each sample was polished, and carbon dust was evenly spread on the polished surface to aid in visualizing the porosity. Then, cavities on the sample surface were observed using an optical microscope (SZ-PT, Olympus Co., Japan), and an image of the sample was captured. The image was analyzed using Image-Pro Plus 7.0 software (Image-Pro; Media Cybernetics Inc., Bethesda, MD, USA). Porosity observation using an optical microscope was shown in Figure 1.

### 2.3. Compression Test

**Figure 2:**
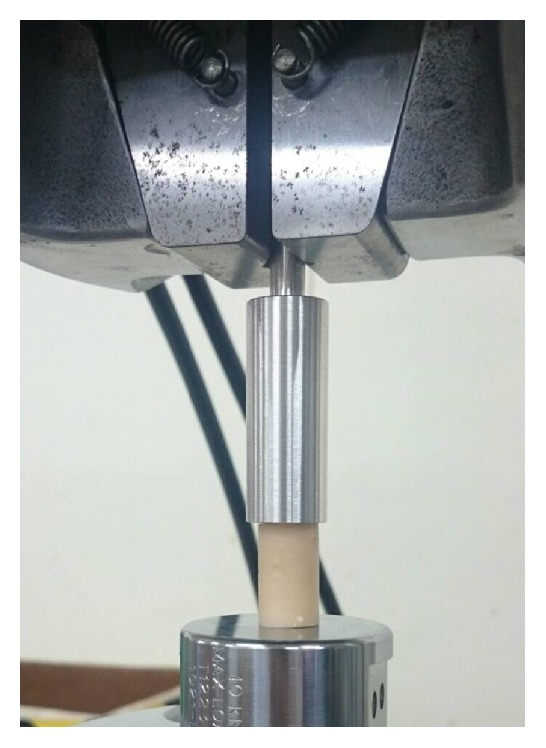
Photograph showing the compression test of cement sample. The specimen was prepared in a cylindrical shape with a 13 mm diameter and 26 mm height. A 20 mm diameter cylindrical rod was used as a plunger and clamped to the upper side of the wedge grip, connecting to an actuator.

Compressive testing was conducted according to ASTM D695 guidelines. Six samples in each group were tested to failure under axial compression using an MTS testing machine (Bionix 858, MTS Corp., MN, USA). Each specimen was prepared in a cylindrical shape with a 13 mm diameter and 26 mm height. A 20 mm diameter cylindrical rod was used as a plunger and clamped to the upper side of the MTS wedge grip, connecting to an actuator. Compressive force was applied at a constant crosshead rate of 1.5 mm/min to test the ultimate compression strength of each prepared PMMA specimen. The ultimate compression strength was defined as the measured ultimate compressive force divided by the area of the PMMA sample's radial surface. The instantaneous relationships between the applied force, displacement, and reaction time were simultaneously recorded in increments of 0.05 mm using MTS TestStar II software. The compression test of cement sample was shown in Figure 2.

### 2.4. Measurement of Temperature Profile

**Figure 3:**
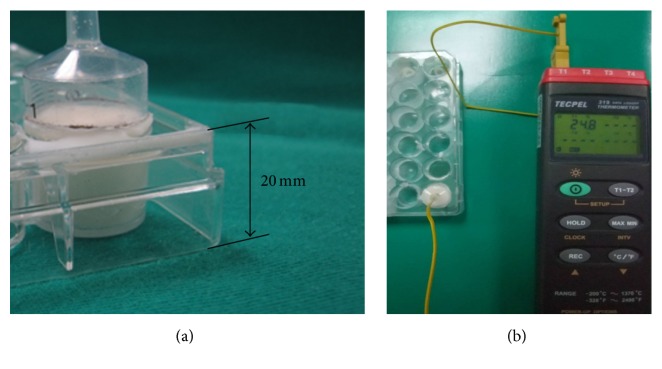
Photograph showing the measurement of temperature profile of cement sample. (a) A cylindrical syringe was cut to a height of 30 mm and used as a container to hold PMMA for measuring temperature profiles. The prepared cement mixture was added to the cavity of the syringe up to 20 mm in height. (b) Then, a thermocouple was inserted into the bone cement to a depth of 10 mm.

A cylindrical syringe with a diameter of 16 mm was cut to a height of 30 mm and used as a container to hold PMMA for measuring temperature profiles. The prepared samples were divided into two major groups, NTG and PCG, with four subgroups (six samples in each subgroup). PMMA was prepared following the same process described above. After the polymer powder and liquid monomer were mixed, the mixture was added to the cavity of the syringe up to 20 mm in height (Figure 3(a)). A height of 20 mm was chosen because it was determined to be similar to that of the vertebral body. Then, a thermocouple (DTM319, Tecpel Co., Taiwan) was inserted into the bone cement to a depth of 10 mm (Figure 3(b)). The temperature change in the center of each sample was measured, and the setting temperature (*T*_set_) was calculated using the equation *T*_set_ = (*T*_max_ + *T*_amb_)/2 following ASTM-F451 specifications (*T*_amb_: ambient room temperature, 22°C) [23]. The handling time (HT) was defined as the duration from the start of mixing to *T*_set_.

### 2.5. Statistical Analysis

All of the measurements were collected in six trials and are expressed as the mean ± standard deviation (SD). Nonparametric Mann–Whitney *U* test was performed to evaluate difference among groups. Differences were considered significant at *P* < 0.05.

## 3. Results

### 3.1. Maximum Polymerization Temperature and Handling Time

**Figure 4:**
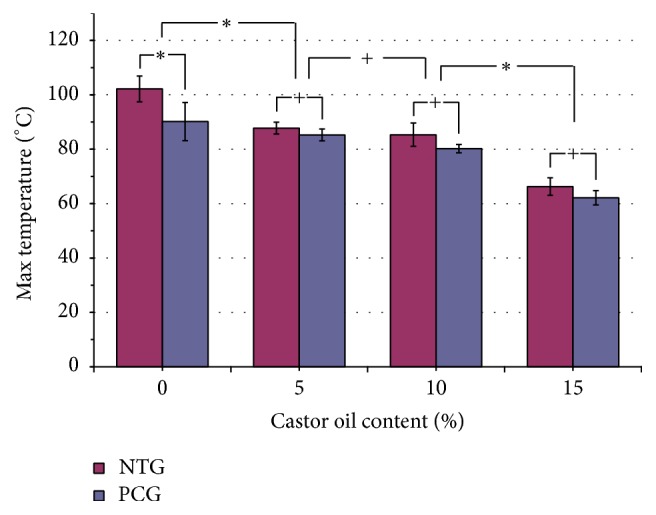
Average maximum polymerization temperature (*T*_max_) for bone cement samples with various contents of castor oil in the NTG and the PCG. The maximum polymerization temperature decreased with increasing castor oil content. However, for a given castor oil concentration (M5, M10, or M15), no significant differences were found between the PCG and the NTG, except for the standard PMMA samples (M0). ^*∗*^ *P* < 0.05. ^+^ *P* > 0.05.

**Figure 5:**
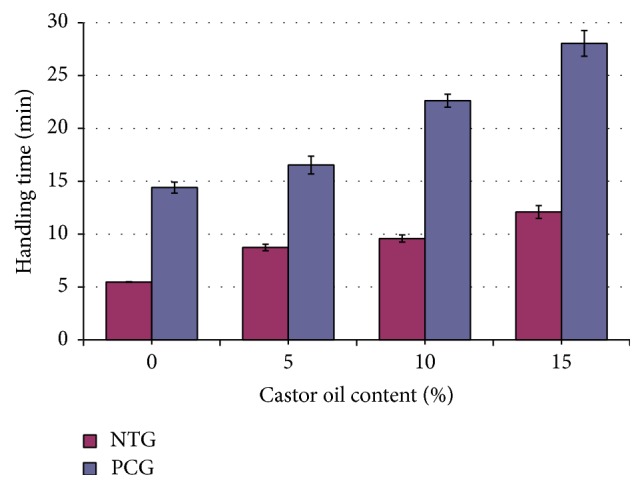
Average HT for bone cement samples with various contents of castor oil in the NTG and the PCG. The HT significantly increased as the castor oil content increased for both the NTG and the PCG. The precooling treatment group exhibited an even greater increase in HT. Significant differences (*P* < 0.05) were found among the groups.

The average maximum polymerization temperatures (*T*_max_) and handling times (HT) for the samples with various contents of castor oil in the NTG and the PCG are shown in Figures 4 and 5. The results showed that the maximum polymerization temperature decreased with increasing castor oil content (Figure 4). In the normal temperature group (NTG-M0 to NTG-M15), the maximum temperature (*T*_max_) decreased by 35.82% between the NTG-M0 (102.18 ± 3.87°C) and NTG-M15 (65.58 ± 2.76°C) samples (*P* < 0.05). In the precooling group, a similar trend was observed. Between the PCG-M0 (93.28 ± 13.91°C) and PCG-M15 (60.28 ± 2.79°C) samples, *T*_max_ declined by 35.4%. However, there were no significant declines between the M5 and M10 groups in either the NTG or the PCG (*P* > 0.05). The handling time (HT) was prolonged in all groups (Figure 5). Further analysis of the NTG and the PCG showed that the HT significantly increased as the castor oil content increased. There was a 121.6% increase (*P* < 0.05) between the NTG-M15 (12.10 ± 0.61 min) and the NTG-M0 (5.46 ± 0.03 min) samples. Similarly, HT exhibited a 94.4% increase between the PCG-M15 (14.40 ± 0.52 min) and the PCG-M0 (28.03 ± 1.22 min) samples (*P* < 0.05). Furthermore, the precooling treatment group exhibited an even greater increase in HT. The increase in time ranged from 1.89-fold in the PCT-M5 (16.53 ± 0.85 min) and NTG-M5 (8.74 ± 0.31 min) samples to 2.64-fold in the PCG-M0 (14.40 ± 0.52 min) and NTG-M0 (5.46 ± 0.03 min) samples. The handling time increased from 5.45 min (NTG-M0) to 28.03 min (PCG-M15).

**Figure 6:**
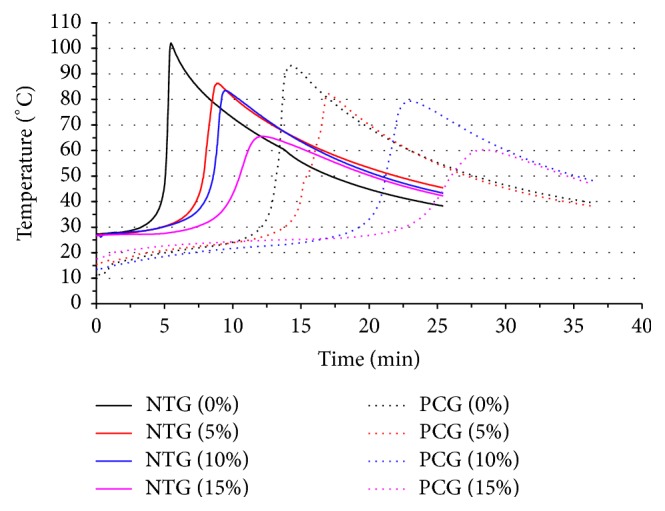
Typical temperature profiles for bone cement samples with various contents of castor oil in the NTG and the PCG. Increasing castor oil content and precooling treatment effectively decreased the peak polymerization temperatures and increased the duration to achieve the peak polymerization temperature.

Precooling treatment also reduced *T*_max_. *T*_max_ declined by 11.79% between the NTG-M0 (102.18 ± 4.77°C) and PCG-M0 (90.15 ± 7.01°C) samples (*P* < 0.05). However, no significant differences were found in the other groups following the addition of castor oil (M5, M10, or M15) (*P* < 0.05). The typical temperature profiles corresponding to the samples with various concentrations of castor oil in the NTG and the PCG are shown in Figure 6. Overall, it was demonstrated that the addition of castor oil and the use of a precooling procedure could decrease *T*_max_ and increase HT.

### 3.2. Biomechanical Evaluation

**Figure 7:**
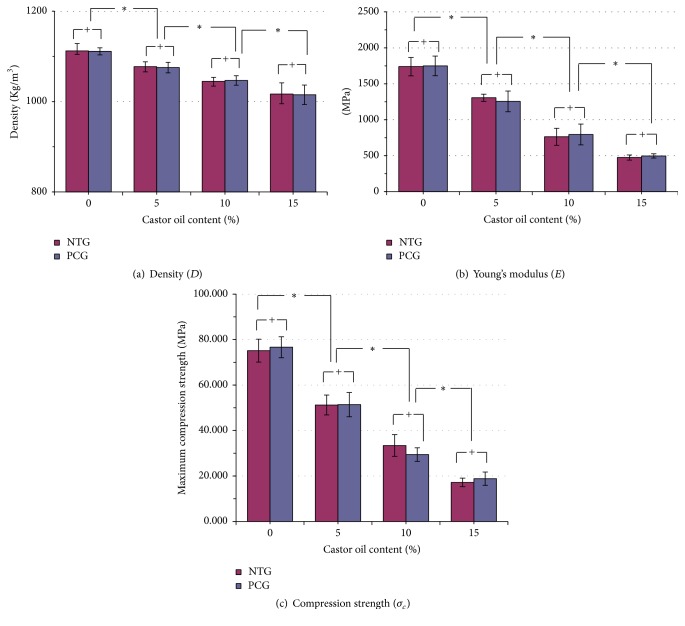
Average (a) density, (b) Young's modulus, and (c) maximum compressive strength for bone cement samples with various contents of castor oil in the NTG and the PCG. The listed properties decreased with increasing castor oil content. However, preparation temperature (room temperature or precooling) had no significant effect (*P* > 0.05) on these properties. ^*∗*^ *P* < 0.05. ^+^ *P* > 0.05.

The average densities, Young's moduli, and compressive strengths for the samples with varying contents of castor oil in the NTG and the PCG are shown in Figure 7 and Table 1. PMMA density decreased with increasing castor oil content. There was an 8.4% decline (*P* < 0.05) between the NTG-M15 (1,017 ± 24.6 kg/m^3^) and NTG-M0 (1,112 ± 15.9 kg/m^3^) samples; similar results were observed in the PCG samples. However, no significant differences were noted between the NTG and the PCG in terms of densities at varying castor oil concentrations. Thus, preparation temperature had no effect on PMMA density.

In regard to the Young modulus (*E*) and the compressive strength (*σ*_*c*_) of each sample, no significant differences were noted between the PCG and the NTG at any castor oil percentage (*P* > 0.05). The precooling treatment had no effect on *E* or *σ*_*c*_ in any of the castor oil groups. Conversely, as the castor oil percentage increased, *E* and *σ*_*c*_ clearly decreased. *E* showed a 72.7% decline (*P* < 0.05) between the NTG-M15 (1,739 ± 128 MPa) and NTG-M0 (474 ± 35 MPa) samples. Similarly, there was a 71.7% decrease (*P* < 0.05) between the PCG-M15 (1,749 ± 137 MPa) and PCG-M0 (495 ± 30 MPa) samples. The results from the compression test also showed large declines in both the NTG and the PCG. For the NTG, the compressive strength decreased by 77.3% between the NTG-M15 and NTG-M0 (*P* < 0.05) samples, whereas the compressive strength decreased by 75.3% between the PCG-M15 and PCG-M0 samples in the PCG (*P* < 0.05).

**Figure 8:**
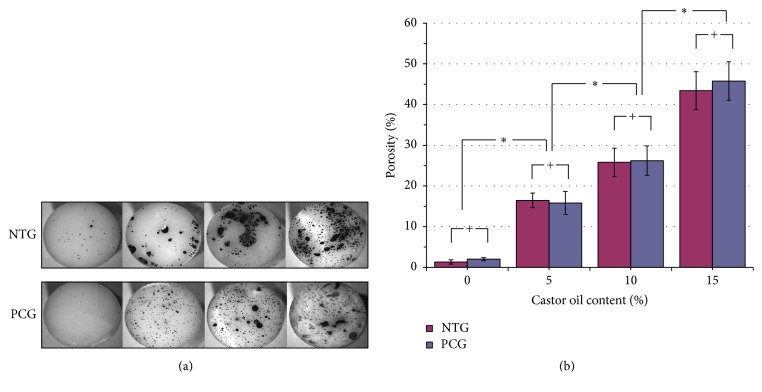
Photograph showing (a) the porous distributions in the bone cement samples and (b) the average porosities for the bone cement samples with various contents of castor oil in the NTG and the PCG. The porosity content increased with increasing castor oil content. However, preparation temperature (room temperature or precooling) had no significant effect (*P* > 0.05) on porosity. ^*∗*^ *P* < 0.05. ^+^ *P* > 0.05.

The porosity distributions and average porosity percentages of the bone cement samples containing varying concentrations of castor oil in the NTG and the PCG are shown in Figures 8(a) and 8(b), respectively. The porosity of PMMA significantly increased as the concentration of castor oil increased in both the NTG and the PCG. In the NTG, the porosity in the NTG-M15 samples (43.4 ± 4.7%) increased by 33.4-fold (*P* < 0.05) compared with that in the NTG-M0 samples (1.3 ± 0.5%), whereas the porosity in the PCG-M15 samples (45.8 ± 6.9%) was 22.9 times (*P* < 0.05) greater than that in the PCG-M0 samples (2.0 ± 0.4%). Similar to the results of the above studies on biomechanical properties, no significant differences in terms of porosity were observed in relation to castor oil content in either the NTG or the PCG.

## 4. Discussion

In this study, the addition of castor oil to PMMA significantly altered the biomechanical properties of PMMA. The addition of castor oil changed all of the measured properties in PMMA, leading to a lower *E*, a lower *σ*_*c*_, a lower *T*_max_, a longer HT, and a higher porosity. In contrast, precooling treatment resulted in a lower *T*_max_ and a longer HT but had no effect on the mechanical properties of PMMA, including *E*, *σ*_*c*_, and porosity.

Obtaining reduced *E* and *σ*_*c*_ in PMMA is an important aim in vertebroplasty. Some studies have indicated that the high *E* of PMMA leads to a risk of causing secondary fractures in neighboring vertebral bodies [17, 18]. The rigidity of traditional PMMA induces local peak stress concentrated near neighboring vertebral bodies after vertebroplasty. This phenomenon may increase the risk of secondary fracture [15, 28]. Although this has not been conclusively proven, it is still a point worth noting. Low-modulus PMMA can more closely match the properties of cancellous bone and exhibits a rigidity similar to that of vertebral bodies after vertebroplasty, as was shown in the NTG-M15 and PCG-M15 samples. However, recent studies have indicated that the target *E* value after vertebroplasty should be closer to that of healthy bone rather than to that of cancellous bone [29]. Thus, in PMMA, having *E* that is similar to that of cancellous bone is not sufficient. Therefore, the properties of the samples created in this study should be compared to those of healthy vertebral bone. In this study, reductions in *E* and *σ*_*c*_ had a linear relationship with the volume of added castor oil. Thus, the *E* and *σ*_*c*_ values of PMMA can be controlled. When a surgeon treats a patient with a vertebral fracture, using the techniques described here, the surgeon could theoretically customize *E* of PMMA to match the bone mineral density of the patient. This process may reduce the risk of secondary fracture after vertebroplasty. In addition, the porosity of the created PMMA samples was also dependent on the amount of castor oil used. The PMMA exhibited higher porosity as higher concentrations of castor oil were used. In a previous study, reduced porosity was observed to produce higher *E* and *σ*_*c*_ values. The porosity resulting from the mixing process used might lead to minor fissures in PMMA. These fissures cause the PMMA less rigid, which may reduce the risk of refracture at the weaker side [28].

The addition of castor oil and the use of a precooling treatment can significantly reduce the temperature of polymerization and prolong the curing time of a sample. In the present study, the maximum reduction in *T*_max_ was a 41.01% decrease, from 102.18 ± 3.87°C in the NTG-M0 samples to 60.28 ± 2.79°C in the PCG-M15 samples (Figure 4). A lower *T*_max_ can reduce the risk of thermal injury to neighboring tissues. However, for castor oil concentrations of M5, M10, or M15, no significant differences were found between the PCG and the NTG. This was not true for the standard PMMA samples (M0), as the castor oil in the PCG was not cooled prior to the experiments; thus, the results were not influenced. For the given concentrations of castor oil, although no significant differences were noted in *T*_max_ between the PCG and the NTG (except in the M0 samples), HT was significantly prolonged between the groups (Figures 5 and 6). These results demonstrated that the precooling treatment had a greater effect than the addition of castor oil with regard to HT. However, for a given concentration of castor oil, the precooling treatment had little impact on the reduction of *T*_max_. Achieving a prolonged HT is important because it provides surgeons with additional time during vertebroplasty. Vertebroplasty can thus be completed more carefully, and further complications can be avoided. In contrast to previous studies [24, 25], our experiments showed that precooling only affects the *T*_max_ and HT of PMMA. Precooling had no influence on the other biomechanical properties tested and produced a synergistic effect when castor oil was added. Thus, physicians can independently control the biomechanical properties of PMMA by selecting the appropriate castor oil volume and can alter the precooling temperature to achieve the required handling time. Overall, our results demonstrated that precooling has a more powerful effect on *T*_max_ and HT than the addition of castor oil. Thus, surgeons can control handling time and decrease potential thermal injury using precooling treatment alone. Furthermore, the presence of porosity was shown to be the main determinant of *E* and *σ*_*c*_ for PMMA. Thus, increasing the porosity of PMMA in a controlled manner is important. The porosity of PMMA could be increased using different mixing techniques and via the addition of different substances. Therefore, future experiments evaluating different methods of increasing porosity via different mixing techniques while the percentage of castor oil is kept constant are warranted.

Our study has limitations. First, specimens prepared under a laboratory environment do not necessarily represent real clinical circumstances. The cement was cured in air without the perfusion of blood, and the environment that PMMA was exposed was not the same as that of living human vertebrae. The possible effects of the variations in the above-mentioned factors were not considered. Second, only one type of bone cement was used. PMMA from different manufacturers may have varying thermal and mechanical properties. Third, our measurements did not take into account the irregular geometry of actual human vertebrae, which may have an impact on the results of all measured parameters. Finally, only static loading (compression tests on bone cement) was used; other types of physiological loading were not considered. In actual clinical situations, PMMA is subjected to dynamic multidirectional loading. Although our loading mode did not necessarily represent actual physiological loading conditions, all of the specimens were prepared and tested in a uniform and reproducible manner, and we believe that this study provides information that could be useful for orthopedic surgeons who perform vertebroplasty. Further investigation into the effects of other loading methods, such as dynamic fatigue testing, might be necessary in the future.

## 5. Conclusion

In the current study, the addition of castor oil and the use of a precooling treatment enabled the creation of PMMA samples with improved biomechanical properties, including lower *T*_max_, longer HT, lower *E*, and lower *σ*_*c*_ values as well as increased porosity. These properties were similar to those of healthy bone; thus, the modified PMMA samples are more suitable for use in vertebroplasty. However, further research is required regarding the use of these PMMA mixtures and their effects on rates of secondary fracture in clinical settings.

## Figures and Tables

**Table 1:** Biomechanical properties of bone cement mixed with varying concentrations of castor oil (0%, 5%, 10%, and 15%) at 25°C and 3°C.

Group		Biomechanical properties			
Treatment	Castor oil (%)	Density (kg/m^3^)	Porosity (%)	*E* (MPa)	*σ*_*c*_ (MPa)
Normal (22°C)	0%	1112.4 ± 15.9	1.3 ± 0.5	1739.4 ± 128.2	75.3 ± 5.0
	5%	1077.6 ± 10.9	16.5 ± 1.8	1306.1 ± 48.8	51.2 ± 4.4
	10%	1044.8 ± 8.9	25.8 ± 3.5	763.3 ± 116.6	33.6 ± 4.8
	15%	1016.8 ± 24.6	43.4 ± 4.7	474.0 ± 35.3	17.2 ± 1.9

Precooling (3°C)	0%	1111.3 ± 7.7	2.0 ± 0.4	1749.1 ± 137.4	77.4 ± 4.6
	5%	1075.4 ± 11.7	15.8 ± 2.8	1255.4 ± 144.1	51.1 ± 5.3
	10%	1046.8 ± 10.4	26.2 ± 3.6	795.3 ± 145.5	29.3 ± 3.0
	15%	1015.1 ± 21.6	45.8 ± 6.9	495.4 ± 30.1	19.1 ± 2.9

Ref. data for cancellous bone [16]				325 ± 145	2.5 ± 1.5

*E*: Young's modulus.

*σ*_*c*_: compression strength.
